# Fine-tuning delivery of cardiac resynchronization therapy: Optimization for “triple fusion”

**DOI:** 10.1016/j.hrcr.2021.03.019

**Published:** 2021-04-08

**Authors:** Niraj Varma

**Affiliations:** Cleveland Clinic, Cleveland, Ohio

**Keywords:** CRT, ECGI, LBBB, LV fusion, Right bundle branch, Triple fusion

## Introduction

Maximizing efficacy and avoiding nonresponse are important aims of cardiac resynchronization therapy (CRT) delivery. Current recommendations emphasize careful candidate selection and left ventricle (LV) lead delivery but do not extend to postimplant optimization in the absence of convincing benefit. However, optimizing electrical resynchronization depends on the interaction between patient substrate and paced effects and may need individualization. Recent data indicate that “Triple Fusion” optimizes CRT delivery.^1^^,^^2^ However, this strategy has never been visually depicted.

**Key Teaching Points**

• Most devices that deliver cardiac resynchronization therapy (CRT) are left at nominal settings after implant, since guidelines do not advocate for routine postimplant optimization in the absence of convincing benefit. However, ventricular activation patterns during intrinsic conduction and responses to right ventricular (RV) and left ventricular (LV) pacing vary among different patients. Thus optimizing electrical resynchronization may need individualization according to the interaction between patient substrate and paced effects.
• Automatic device-based algorithms are appealing solutions for optimization. However, a one-size-fits-all algorithm may not deliver optimal resynchronization in all patients. For example, simply using LV pacing may not resolve large transseptal delays that account for delayed LV activation in left bundle branch block.
• Recent reports indicate that biventricular pacing at individualized atrioventricular intervals is more likely to deliver best electrical resynchronization. This promotes synergy among intrinsic right bundle branch conduction and RV and LV paced wavefronts. This is conceptualized to promote confluent LV activation. This case using electrocardiographic imaging visually depicts this resynchronization scheme of “triple fusion.”
• Currently enrolling randomized trials are testing advantages of electrocardiographic-guided CRT optimization with a device-based algorithm designed to deliver triple fusion therapy (SyncAV; ClinicalTrials.gov Identifier: NCT04100148).

## Case report

A 72-year-old woman with hypertension, diabetes, and recurrent heart failure found to have nonischemic cardiomyopathy, left bundle branch block (LBBB) (QRSd 180 ms), and persistent LV dysfunction (LV ejection fraction 20%) despite guideline-directed medical therapy received CRT with a posterolateral LV position. Thus the patient met best selection criteria (typical LBBB morphology and QRSd >150 ms) and implant technique.

**Figure 1:**
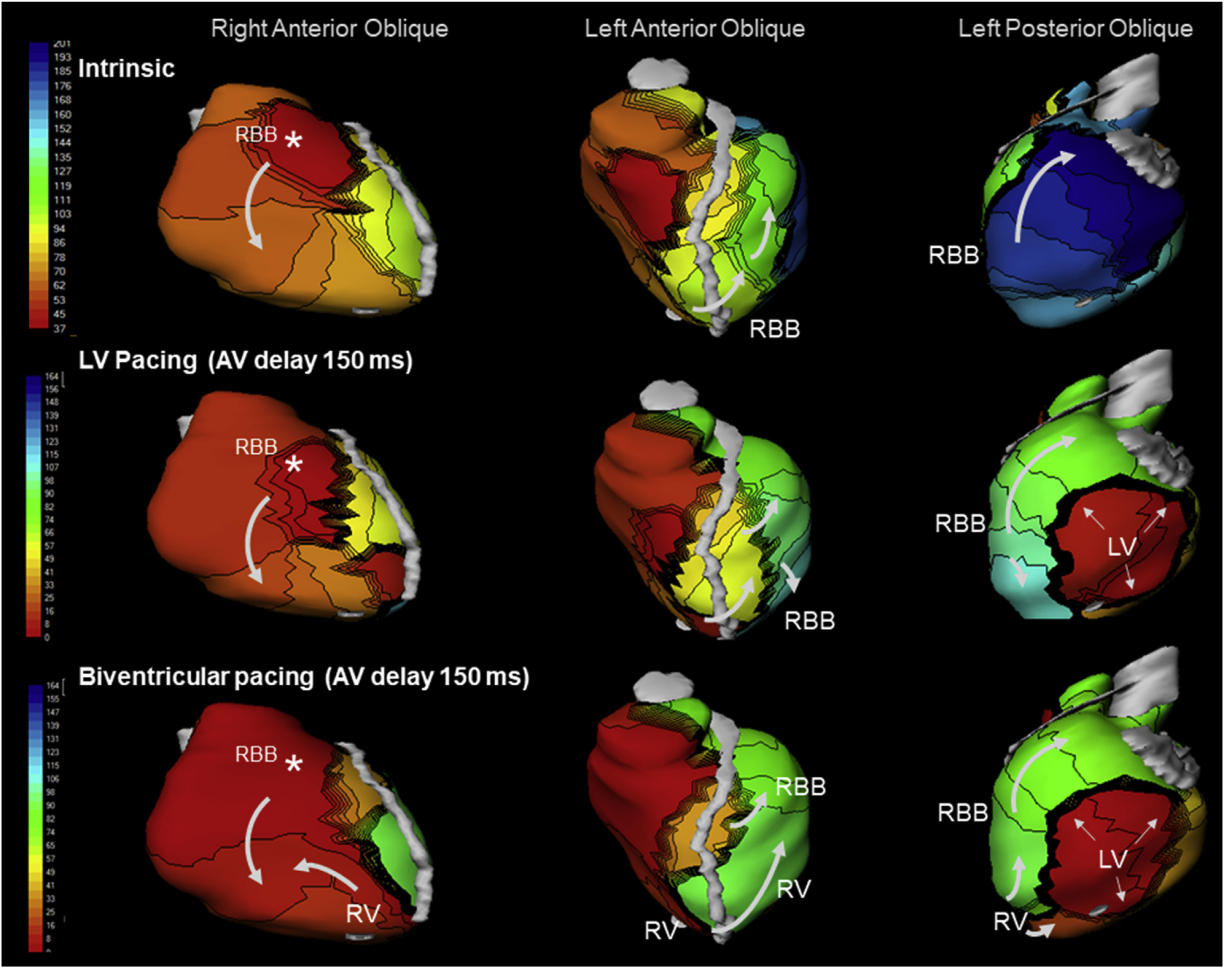
Electrocardiographic imaging presenting 3 different programmed modes in 3 views: first column: right ventricle (RV) free wall; middle column: septum and left anterior descending artery (*gray*); third column: left ventricle (LV) free wall. **Top row:** Intrinsic conduction. (Left) RV activation commences in the free wall (∗) and activation proceeds, typical for heart failure patients with left bundle branch block.^4^ (Middle) Slow conduction across the septum marked by isochronal crowding. (Right) Following this delay, there is late (*dark blue*) but swift LV free wall activation (widely spaced isochrones) finishing 180 ms after the RV. **Middle row:** LV-only pacing. RV and septal activation is unchanged vs intrinsic (left and middle). LV pacing preexcites LV but the propagating wavefront is then limited by functional conduction slowing (*thick black lines*) in regions not vulnerable to delay during intrinsic conduction. This has a functional basis, since these areas were not observed during intrinsic conduction (above). Hence, although LV pacing resulted in significant LV preexcitation (*red*) this was limited in area—the remainder of the LV was depolarized by the late effects of right bundle branch (RBB)-mediated LV activation. **Bottom row:** Biventricular pacing. RBB and RV paced wavefronts synergize to facilitate RV depolarization (left). RV pacing accelerates inferoapical transseptal activation (middle) and inferoapical LV depolarization (ie, regions not preexcited by LV stimulation)—that is, RV pacing improves LV activation. The RBB maintains contribution to anteroseptal depolarization.

**Figure 2:**
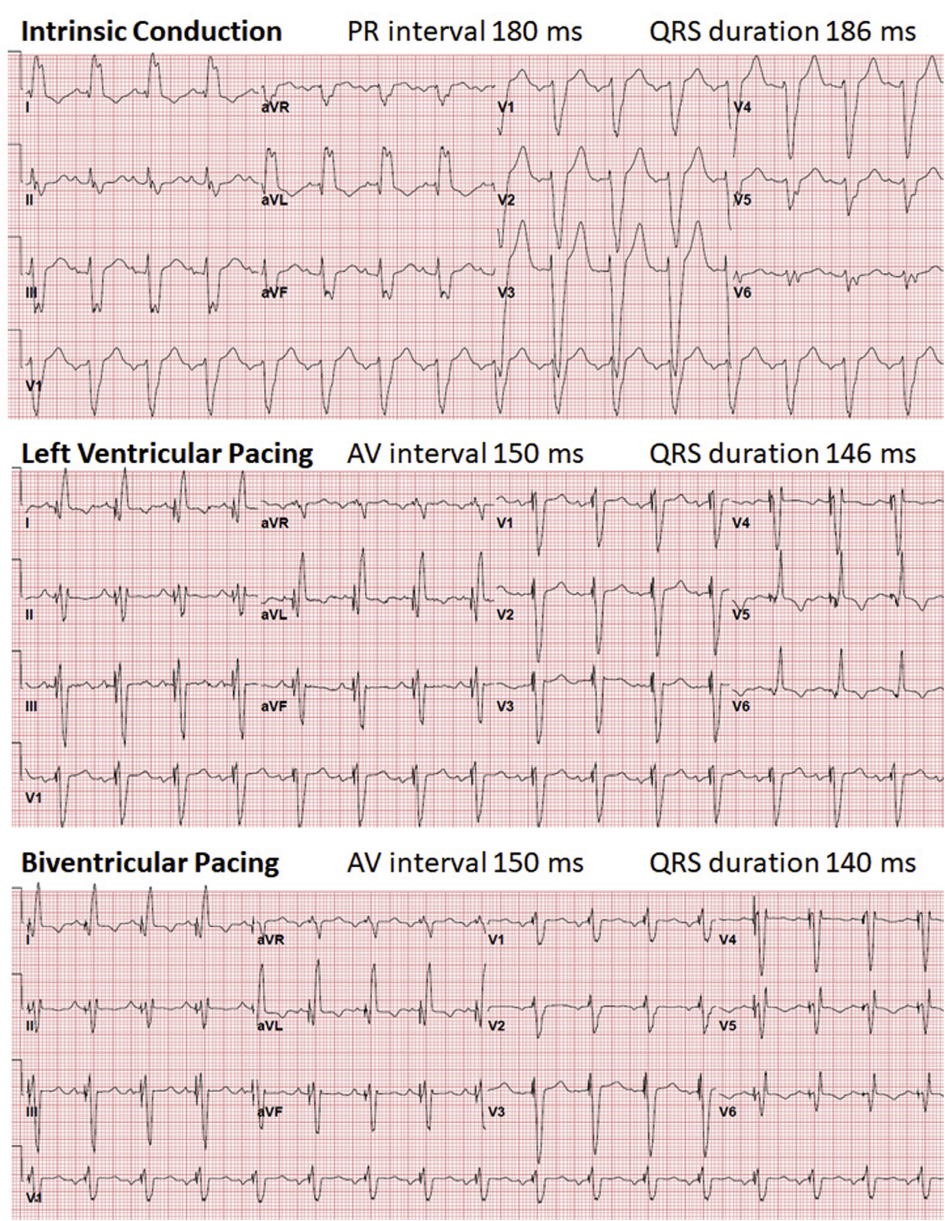
Corresponding electrocardiograms.

Electrocardiographic imaging (ECGI) study was undertaken 1 day post-implant (Figure 1; corresponding electrocardiograms in Figure 2). The methodology has been described previously.^3^^,^^4^ Briefly, ECGI acquires more than 200 channels of body surface electrograms at 1-millisecond intervals during the cardiac cycle using a multielectrode vest. Epicardial geometry and electrode position are registered by computed tomographic scan. Data are processed with algorithms to obtain epicardial potentials, electrograms, and activation sequences (isochrones). Here, activation times and sequences during intrinsic and paced rhythms were examined. Time zero was set at the beginning of QRS for all native rhythm episodes and at ventricular pacing stimuli for all paced episodes.

The ventricular activation underlying LBBB is shown in Figure 1 (top row). Important features are intact right bundle branch (RBB) conduction responsible for normal centrifugal right ventricle (RV) depolarization (left panel).^4^ The RBB is responsible also for LV activation in the absence of LBB conduction. This follows slow transseptal conduction (top middle). LV free wall depolarization begins late but, once initiated, finishes rapidly (top right). The transseptal delay is important, since it determines QRS prolongation, and is a functional barrier that is affected by RV pacing (see below).

LV pacing is the therapeutic element of CRT. However, LV epicardial stimulation elicits wavefronts that propagate slowly even in normal myocardium (demonstrated almost 100 years ago by Carl Wiggers). These vary significantly among patients with LBBB and heart failure and are unrelated to LV activation delay during intrinsic conduction (“qLV”).^3^ Here, LV activation following LV stimulation initially is relatively rapid (middle row, right, red area) but then limited by conduction slowing.

LV paced effects require coordination with intrinsic RBB conduction (“LV-only fusion pacing”) and/or RV pacing (biventricular pacing) to promote biventricular resynchronization. This underlies optimization methods. LV pacing alone timed to fuse with intrinsic RBB conduction promises LV preexcitation while avoiding RV paced electrical delays.^4^ This is appealing and forms the basis of 1 device-based algorithm (AdaptivCRT). However, this showed no advantage in primary trial endpoint compared to biventricular pacing in control patients.^5^ Here we show that this combination does not resolve septal delay (ie, the root problem created by LBBB) or affect activation of the anterolateral and anteroapical LV (middle row, center), and thus *creates* intra-LV electrical dyssynchrony.

Recent reports indicate that biventricular fusion pacing is more likely to deliver better electrical resynchronization than LV-only fusion pacing.^2^ The reason why inclusion of RV pacing is beneficial appears puzzling. However, the RV pacing component of CRT may have an important action on transseptal delay, disintegrating parts of this barrier and preexciting the anteroapical LV (Figure 1, bottom row, middle). Interestingly, this figure panel shows that during biventricular pacing, the contribution of intrinsic RBB conduction to septal and anterolateral LV depolarization is maintained. These wavefronts together result in more confluent LV activation—that is, “triple fusion.” Moreover, although RV pacing is regarded as causing RV activation delays,^5^ here we show the opposite: RV activation was accelerated because intrinsic RBB conduction and RV paced wavefronts synergized (bottom row, left).^4^

This is the first visual depiction of “triple fusion”—that is, effects of intrinsic RBB conduction combining with RV and LV paced wavefronts to promote best biventricular resynchronization.^1^ This requires utilizing the initial RV paced effect (without committing the entire LV to the RV paced wavefront) and AV interval selection to optimize RBB contribution, in concert with LV paced effect. One recent randomized trial showed that, among patients with LBBB, individualized optimization of the atrioventricular (AV) interval with biventricular pacing most often promoted best electrical optimization and elicited an improved degree of CRT response.^2^

This single case report does not reflect the variability in ventricular activation patterns that occur during intrinsic conduction and in response to LV and RV pacing among different patients.^3^ Novel techniques utilizing septal/LBBB area pacing are designed to overcome septal conduction barriers and may thereby facilitate triple fusion. These observations emphasize that programming has to be individualized and triple fusion may not always be able to be delivered (and even may be unnecessary when there is minimal transseptal conduction delay). Further, optimized settings may change with time as the heart remodels with ongoing therapy. Currently enrolling randomized trials are testing advantages of ECGI-guided CRT optimization over time (NCT03504020) and a device-based algorithm designed to deliver triple fusion therapy (SyncAV: ClinicalTrials.gov Identifier: NCT04100148).

This patient was committed to simultaneous biventricular pacing (AV interval 150 ms) to deliver triple fusion. LV function rectified (LV ejection fraction 40% at 1 year and 60% at 18 months post-implant) and has remained stable for 10 years.

## Conclusion

Patient individualized optimization offers opportunity to improve both biventricular and intra-LV synchrony during CRT.
